# Implementing Patient-Directed Cancer Education Materials Across Nigeria

**DOI:** 10.1200/GO.21.00233

**Published:** 2021-12-03

**Authors:** James C. Dickerson, Paulette Ibeka, Itoro Inoyo, Olufolarin O. Oke, Sunday A. Adewuyi, Donna Barry, Abubakar Bello, Olufunke Fasawe, Philip Garrity, Muhammad Habeebu, Franklin W. Huang, Vivienne Mulema, Kenneth C. Nwankwo, Danna Remen, Owens Wiwa, Ami S. Bhatt, Mohana Roy

**Affiliations:** ^1^ Department of Medicine (Hematology and Oncology), Stanford University, Stanford, CA; ^2^ Clinton Health Access Initiative, Inc, Abuja, Federal Capital Territory, Nigeria; ^3^ Department of Medicine, UT Southwestern, Dallas, TX,; ^4^ Oncology, Ahmadu Bello University Teaching Hospital Zaria, Nigeria; ^5^ Global Oncology Inc, Oakland, CA; ^6^ Radiotherapy, Lagos University Teaching Hospital, Idi Araba Lagos, Nigeria; ^7^ Lagos University Teaching Hospital, Idi Araba Lagos, Nigeria; ^8^ Global Oncology Inc, Oakland, CA; ^9^ University of Nigeria Teaching Hospital, Enugu, Nigeria; ^10^ Department of Medicine (Hematology, Blood and Marrow Transplantation) and of Genetics, Stanford Center for Innovation in Global Health, Stanford University, Stanford, CA; ^11^ Global Oncology, Oakland, CA; ^12^ Department of Medicine, Oncology, Stanford University, Stanford, CA

## Abstract

As access to cancer care expands in low-income countries, developing tools to educate patients is paramount. We took a picture booklet, which was initially developed by the nonprofit Global Oncology for Malawi and Rwanda, and adapted it for use in Nigeria. The primary goal was to assess acceptability and provide education. The secondary goals were (1) to describe the collaboration, (2) to assess knowledge gained from the intervention, (3) to assess patient understanding of their therapy intent, and (4) to explore patient's experiences via qualitative analysis.

**METHODS:**

We piloted the original English booklet at a single site and requested feedback from patients and providers. The booklet was updated; translated into Hausa, Yoruba, Igbo, and Pidgin English; and used at three additional sites. For the three-site cohort, we collected basic demographics, pretest and post-test assessing content in the booklet, and performed a qualitative analysis.

**RESULTS:**

The original booklet was widely acceptable and recommended by patients at site one (n = 31) and by providers (N = 26) representing all four sites. In the three-site cohort (n = 103), 94% of patients recommended the booklet. An immediate post-test focusing on when patients should present to care showed a statistically significant improvement in one of the seven questions. Fifty-one percent of the patients (n = 103) knew their treatment intent (curative *v* palliative). Qualitative analysis highlighted that the patient's thoughts on cancer are dominated by negative associations, although curability and modern therapy are also frequently cited.

**CONCLUSION:**

We adapted an educational booklet to a novel context and had it delivered by local partners. The booklet was widely recommended to future patients. The booklet had an impact on patient's knowledge of cancer treatment, potentially allowing for decreased abandonment.

## BACKGROUND

In 2012, Global Oncology, Inc (GO), a United States–based nonprofit organization with the mission to improve patient education in low- and middle-income countries (LMICs), partnered with low-literacy experts and hospitals in Malawi and Rwanda to create a picture-based booklet named *Cancer and You*. The eleven-page booklet focuses primarily on explaining treatment side effects. This is because poor understanding of therapies is often cited as a reason for treatment abandonment, and it has been postulated that improving patient understanding may yield improved compliance.^1-3^ The GO booklet has been used in multiple countries, has been translated to more than 20 languages, and more recently was studied formally in a small pilot in Haiti.^4,5^

**CONTEXT**

**Key Objective**
How do we develop effective cancer education materials that decrease treatment abandonment for patients in low- and middle-income countries? We describe the process of adapting an educational booklet to a novel context in Nigeria and having local partners deliver the intervention.
**Knowledge Generated**
The booklet showed improvement in one of seven questions on the post-test assessing content covered in the booklet.
**Relevance**
We demonstrated that an educational booklet developed for low- and middle-income countries could have impact on patient's knowledge of their cancer treatment, potentially allowing for decreased abandonment.

Here, we describe the largest implementation of the GO picture booklet. This was done via collaboration between multiple US-based universities, GO, the Clinton Health Access Initiative (CHAI), and four cancer clinics in Nigeria. Nigeria provides a unique opportunity to study educational tools, given its multicultural nature: the largest ethnic group represents < 30% of the total population, and each region is distinct in language, education level, and politics.^6^ Nigeria also has < 50 clinical oncologists for its population of roughly 200 million; so, time for robust patient-provider discussions is limited.^7,8^ Given these factors, Nigeria was an ideal location for testing the booklet.

The primary goals of this study were to assess acceptability of the educational tool and provide education to patients regarding symptoms and side effects. The secondary goals were (1) to lay a roadmap for international partnerships, (2) to assess whether patients gained knowledge from the booklet, (3) to explore patient's understanding of their therapy intent, and (4) to characterize the cancer patient experience through qualitative analysis. We collected basic demographic data, pretest and post-test assessing knowledge gained from the booklet, and performed a qualitative analysis of patient's interviews regarding both the booklet and their experience with cancer.

## METHODS

### Collaboration Overview

This project involved continuous collaboration between the universities, GO, CHAI, and four Nigerian cancer centers. In 2017, Stanford University and Global Oncology established a relationship with the Nigerian Ministry of Health and two Nigerian hospitals to support capacity development.^9^ On a site visit to Lagos University Teaching Hospital (LUTH), a multidisciplinary tumor board was modeled with providers from Stanford, LUTH, and Ahmadu Bello University Teaching Hospital (ABUTH). During this meeting, the Nigerian clinicians noted that treatment abandonment was a substantial problem. Given the aim of the GO booklet to decrease abandonment through education on therapy side effects, the Stanford team presented the booklet and all parties discussed how to study it in Nigeria. Representatives from CHAI were also present at this meeting. CHAI supports the Nigerian government with expanding access to cancer care.^10^ Given their local knowledge, this organization was well suited to provide on-the-ground support. CHAI also introduced the Stanford team to oncologists at the other two cancer centers that also participated in the study.

Following the Nigerian government's commitment to support the project, the protocol was developed for review and approved by the Stanford Institutional Review Board. The protocol was also reviewed at each participating hospital. Because the project did not include patient identifiers, it was exempt from the Nigerian Federal Ministry of Health's National Health Research Committee review.

### Booklet Adaptation

**FIG 1:**
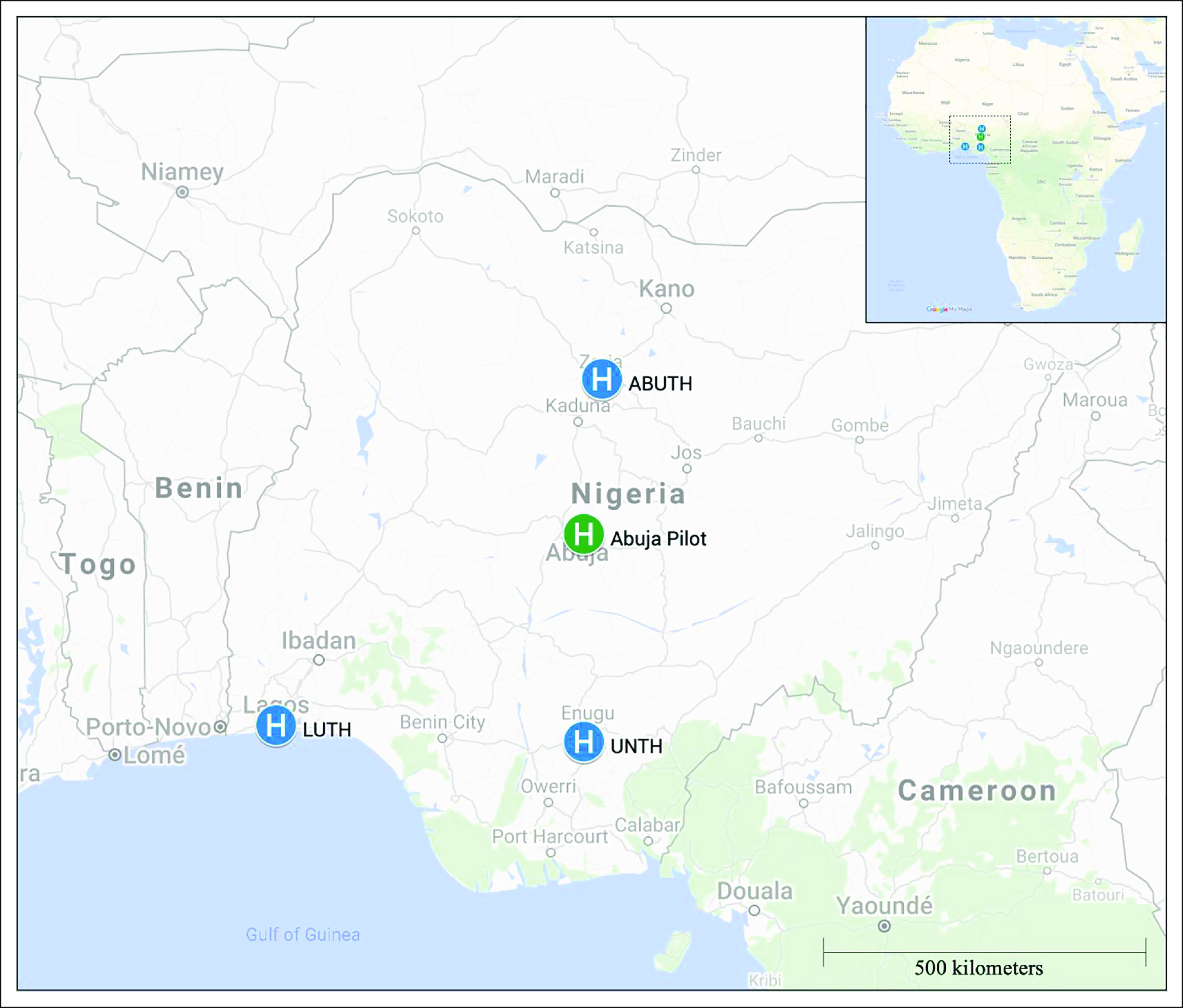
Map with participating clinical sites in Nigeria. Abuja (the capital of Nigeria) is where the pilot occurred and where the main CHAI (Clinton Health Access Initiative) office is located. The subsequent study sites are also indicated on this map, as follows: ABUTH, Ahmadu Bello University Teaching Hospital in Zaria; UNTH, University of Nigeria Teaching Hospital Enugu in Enugu; LUTH, Lagos University Teaching Hospital in Lagos.

The process of creating the GO booklet has been described previously and involved collaboration between GO, Queen Elizabeth Central Hospital (Blantyre, Malawi), Butaro Cancer Center (Butaro, Rwanda), low-literacy experts from the Health Communication Core at the Dana Farber Cancer Institute (Boston, MA), and a design consultancy (The MEME Design; Cambridge, MA).^3^ The original booklet (2017 version) was sent to providers at all four clinics in July 2018. Feedback was obtained via phone interviews from physicians, nurses, and pharmacists at each site. The original protocol (Data Supplement) intended for this provider feedback to be completed before using the booklet with Nigerian patients, although the team experienced significant challenges with scheduling and completing the interviews. Thus, the 2017 English version of the booklet was used with patients at a single first site (National Hospital Abuja [NHA]; Abuja, NG) that was close to the main CHAI office while provider feedback was also being collected (Fig 1). The feedback from providers and patients at NHA informed a booklet update (2019 version; see attachment). This 2019 English version was then translated by professional translators into Hausa, Yoruba, Igbo, and Pidgin English. Quality assurance was done via back-translation to English and having multiple bilingual health care providers review the translated work.

### Data Collection and Analysis for the Three-Site Cohort

At each hospital, the oncology department head was the lead contact. This person nominated a resident physician and a nurse to be the data collectors. The CHAI team (authors P.I. and I.I.) trained the data collectors with a 2-hour, in-person session going over the booklet and questionnaire (see the Data Supplement for questionnaire). The CHAI team supervised most of the data collection.

At each site, a convenience sample of participants was introduced to the concept of the study, provided informed consent, and interviewed before a clinic visit. The interviews were conducted by the data collectors and lasted 30-60 minutes. The pretest was performed orally, after which the booklet was given and explained to the patient in the appropriate language. This was followed by an immediate oral post-test and interview. Interviews were performed daily for a total of 2 weeks at each site. The data recorder wrote down the responses with pen and paper. The first pilot started at NHA in September 2018, and the final interviews were completed at the University of Nigeria Teaching Hospital Enugu (UNTH) in September 2019. At NHA, LUTH, and ABUTH, the CHAI team was present for the interviews, assisted the data collectors, and then entered the data into Excel (Microsoft Corporation, Redmond, WA) at a CHAI office. In Enugu state, where UNTH is located, CHAI has no presence, and so, the data collectors did the interviews independently and then mailed the completed questionnaires to the nearest CHAI office in Rivers state.

The survey questions were selected on the basis of feedback from providers in Malawi and Haiti, and, on the basis of content covered in the original 2017 booklet. For the seven questions asked in both the pretests and post-tests, we calculated averages and used a paired Student's *t*-test to evaluate whether there was a difference in the scores for either (1) education level (split into greater *v* less than secondary education) or (2) booklet language (English *v* other). We used McNemar's exact test to examine improvement with the intervention for matched pre- and post-test questions. We considered a *P* value of < .05 statistically significant. Calculations and analyses were performed in Excel.

### Qualitative Analysis Methods

Patient's responses were written down by hand by the data collector as verbatim as feasible, although there was likely variability among different sites. Then, the CHAI team translated and entered the responses into an Excel database. For the qualitative analysis, we used conventional inductive content analysis.^11^ An inductive approach (ie, creating themes based upon the content reviewed) was chosen, given the limited prior literature on the Nigerian cancer patient experience. Deductive analysis (ie, focusing on how things are said) was limited as all conversations were transcribed and then often translated to English by CHAI.^12^ Recorded responses were imported into NVivo 1.3 (QSR International; Melbourne, Australia). The inductive process included multiple reviews of the raw data, with the primary mode of identifying themes and categories via coding of the responses by two authors (M.R. and J.C.D.). Coded themes were labeled initially by broad categories (eg, symptoms and treatment intent) as guided by the questions, and then subdivided in a hierarchical fashion (eg, respiratory *v* GI symptoms, curative *v* palliative intent, respectively). Both coders generated a similar list of broad categories, and then worked in turn with review of the other's work to maintain consistency in the methodology. The most frequently coded themes are included in the results section, along with quotations that were felt to be representative of the associated themes. Word frequency and the length of recorded responses were also examined.

## RESULTS

### Provider Feedback

Twenty-six providers from all four centers provided feedback on the 2017 version of the booklet. Most of the providers noted that the booklet was easy to understand (77%; 20 of 26), well organized (77%; 20 of 26), and easy to use with patients and caregivers (88%; 23 of 26). All providers recommended the booklet overall.

### Patient Feedback from the Pilot at NHA

Thirty-one patients provided feedback on the 2017 version of the booklet. Demographics and cancer type were only reported by 50% of the patients and so are not included in the manuscript. Patients recommended adding information on nutrition/diet and pregnancy/family planning. Patients also recommended expanding the information on treatment modalities in the booklet. One patient asked for a discussion of treatment intent (palliative *v* curative) and another patient asked for the cost of care and how to acquire financial assistance. All patients (n = 31) recommended the booklet.

On the basis of this feedback, the following was added to the 2019 version of the booklet: additional content on how cancer is treated, what curative versus palliative intent means, cancer and pregnancy, and a section on maintaining medical contact. This 2019 version was used in the expanded three-site cohort. The changes that were made to the booklet are highlighted in yellow in the attached Data Supplement.

### Expanded Three-Site Cohort Demographics

**FIG 2:**
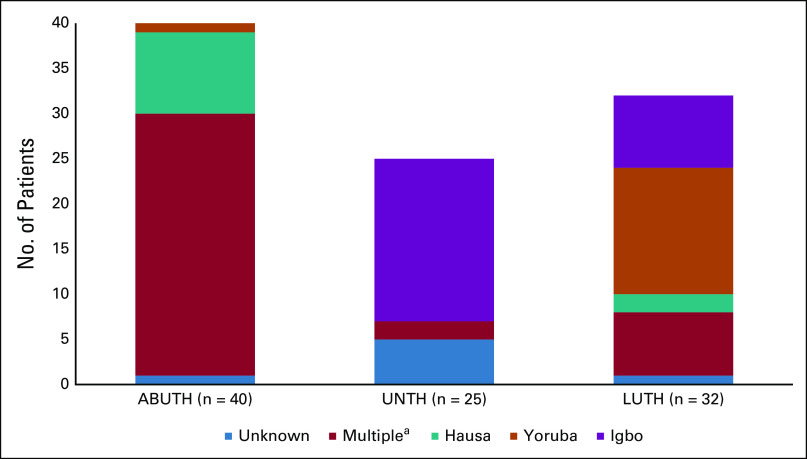
Ethnic representation by clinic site. The figure shows self-reporting ethnic group representation by treatment site. ^a^Multiple includes the following ethnic groups of which there were ≤ 3 representatives: Afo, Atyap, Bajju, Baruba, Bata, Bini, Boki, Dakarkari, Delta, Edo, Fulani, Fulfude, Gwadira, Idoma, Igala, Ikulu, Ikwerre, Jaba, Jinye, Jukun, Kanuri, Kare-kare, Michika, Nizun, Nupe, Tangale, Tiv, Ubibio, and Yungur. ABUTH, Ahmadu Bello University Teaching Hospital in Zaria; LUTH, Lagos University Teaching Hospital in Lagos; UNTH, University of Nigeria Teaching Hospital Enugu in Enugu.

**FIG 3:**
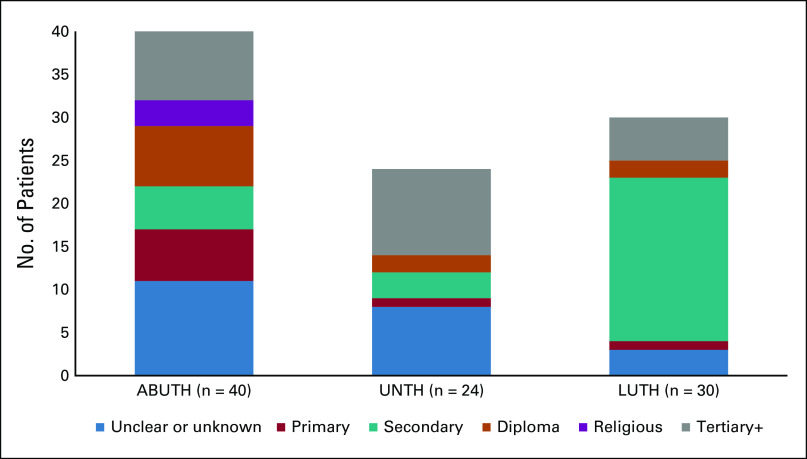
Education level by site. The figure shows the available self-reported education levels of the patients at each site. It is unclear if the unknown was an attempt to record no formal education or a data entry error. The category of diploma includes the following recorded responses: diploma, Nigeria certificate in education (NCE), higher national diploma (HND), and ordinary national diploma (OND). The category of religious degrees is heterogeneous and may represent training after completion of secondary school or a religious education in lieu of another primary and secondary education. For the tertiary+ category, about half of the category had a university degree and the other half a masters. One patient at UNTH had a PhD. ABUTH, Ahmadu Bello University Teaching Hospital in Zaria; LUTH, Lagos University Teaching Hospital in Lagos; UNTH, University of Nigeria Teaching Hospital Enugu in Enugu.

The three-site cohort (n = 103) was 76% female, the mean age was 51 ± 14 years, 75% of patients were married, and, of the 75 patients with a documented education level, 88% had a secondary education or higher. Representatives from 32 ethnic groups (n = 90) were included in the sample. Only three ethnic groups had more than three representatives: Igbo (n = 26), Yoruba (n = 15), and Hausa (n = 11). Each site had a large variation in ethnicity (Fig 2) and education level (Fig 3). Cancer types are reported in Table 1. Eighty-seven percent (90 of 103) of the patients used the English language booklet. Two sites (ABUTH and UNTH) exclusively used the English booklet. At LUTH (n = 29), 67% used English, whereas 19% used Yoruba, 14% used Pidgin, and 1% (one patient) used Igbo. No patients used the Hausa booklet.

**TABLE 1:**
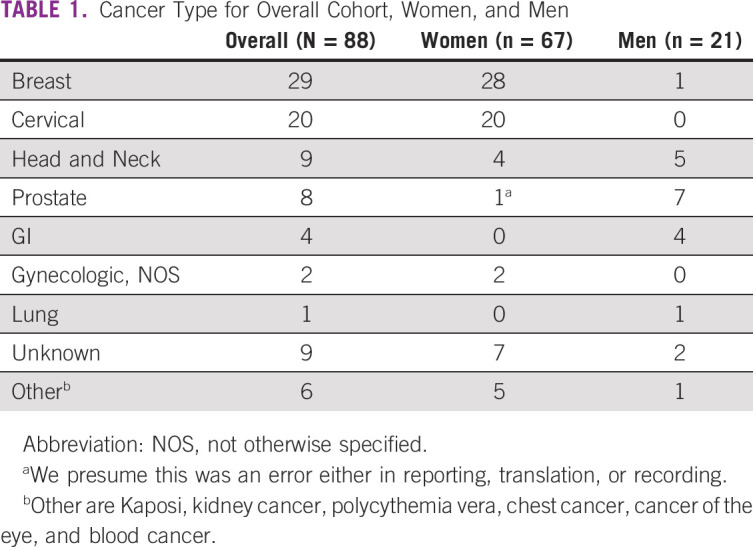
Cancer Type for Overall Cohort, Women, and Men

### Three-Site Survey Results

Most (72% of subjects) found that the booklet answered their questions, and 93% of subjects would recommend it to other patients with cancer (Table 2). This was true regardless of the booklet language used. There was high concordance between the pretest and post-test, with most patients knowing the correct answers before the intervention. In comparing pre-/post-test scores, one of the seven questions for the overall group showed a significant improvement after the intervention (“Should you stop chemotherapy if you have other medical conditions like HIV/AIDS, TB, or diabetes?”; Table 2). There was no difference in pretest versus post-test scores when we split the data by education level or booklet language.

**TABLE 2:**
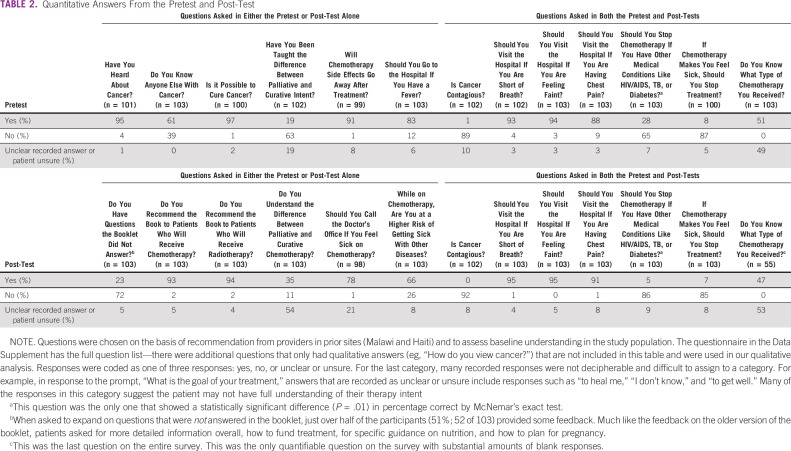
Quantitative Answers From the Pretest and Post-Test

Fifty-one percent of the patients knew their own therapy intent when asked in the pretest (Table 2). In the pretest, 49% (50 of 103) of patients clearly reported that they thought they were receiving curative therapy and 2% (2 of 103) reported receiving palliative chemotherapy (similar in the post-test). Data on provider-reported therapy intent, cancer stage, and date of diagnosis were not available.

### Three-Site Qualitative Analysis Results

**FIG 4:**
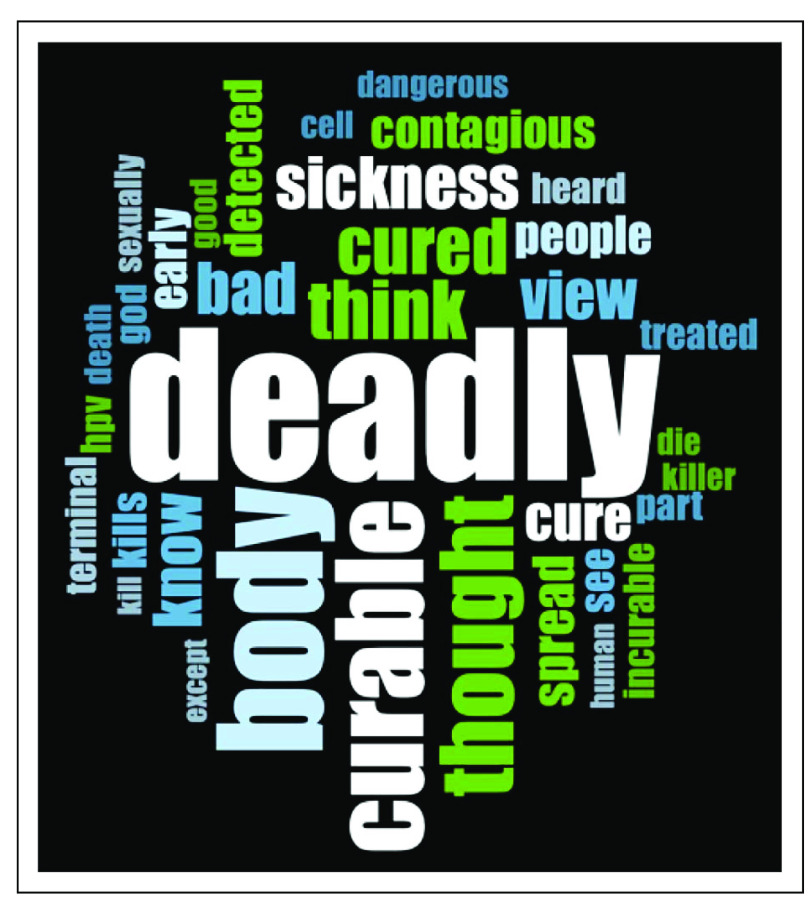
Word cloud of the most frequent words recorded for thoughts on cancer. We took the recorded responses from a prompt “What are your thoughts on cancer?” and examined word frequency from the pooled responses. The largest words in the cloud are the frequent ones: deadly (41), body (28), curable (23), thought (19), think (15), cured (14), bad (13), and sickness (13).

For the overall cohort, the most frequently used words are shown in Figure 4. The recorded responses became shorter over the course of the survey, going from 3,000-4,000 characters on average in the first half of the survey to around 1,500-2,500 characters in the second half of the survey. Identified themes included the deadly nature of cancer, the curability of cancer, fear of the disease, and the cost of cancer and treatment (Table 3). Responding to a prompt of “How do you treat cancer outside of the hospital?,” there was a great diversity of responses, with the most frequently mentioned items (mentioned by around one third of patients) being traditional or herbal treatments and food or nutrition.

**TABLE 3:**
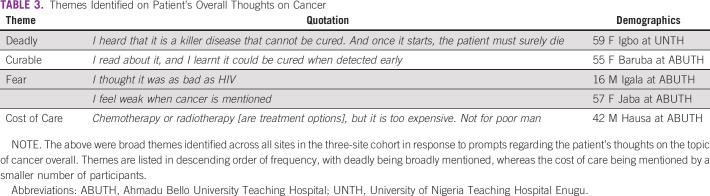
Themes Identified on Patient's Overall Thoughts on Cancer

## DISCUSSION

We describe the adaption and implementation of a cancer education booklet in Nigeria. The goals were to assess acceptability, educate patients, describe this collaborative process as a roadmap for future work, investigate the booklet's impact on knowledge, explore patient's understanding of therapy intent, and characterize the Nigerian cancer experience through qualitative analysis. We found that the booklet was highly recommended by our cohort of patients and providers, indicating acceptability among a diverse group of patients and providers. It was able to improve performance on an immediate seven-question post-test focused on when to present to care. We also found that the highly educated cohort had confusion about the intent of their therapy and displayed negative emotions toward cancer.

Adapting the booklet for Nigeria provided several practical lessons. Although the Nigerian Federal Ministry of Health oversees health care generally, each tertiary center is independent in its operation. Hence, there was duplicate work involved with engaging each hospital administration. This was largely done by CHAI and contributed to about a 6-month delay. Having an in-country support team (in CHAI) that was familiar with the Nigerian health care system, and able to assist at multiple sites, was invaluable. Partnering with providers at every clinic was critical to study completion, and we want to emphasize the importance of these relationships.

Having the clinics directly perform the data collection reinforced the collaboration and removed the introduction of an external interviewer. We decided to have the data collectors write down answers, which was quick and more cost-effective, but led to likely errors in data entry (eg, a female with prostate cancer). It also severely constrained the qualitative analysis. There was likely fatigue from both the interviewer and interviewee as recorded responses became shorter and shorter over the duration of the survey. In future studies, it may be preferable to streamline the survey to improve the completeness of data collection. Finally, recording and transcribing may be preferable for future investigations, despite the fact that this can be expensive and cumbersome.^13^

Our patient population skewed female, and breast cancer and cervical cancer were the most reported pathologies. These demographics are consistent with both our experience and the available registry data.^14,15^ It is worth noting that significant efforts are underway to work on breast cancer screening, HPV vaccinations, and cervical screening in Nigeria (Cervical Cancer-Free Nigeria campaign).^16-19^

The acceptability of the booklet in this study was similar to the implementation experience in Rwanda and the prior pilot in Haiti.^4,5^ Comparing the Haitian and Nigerian studies highlights a key difference in education level: 88% of the Nigerian sample (with a known education level) had a secondary education level or higher in comparison to only 40% (8 of 20) in the Haiti study. The pretest score average was about 50% in Haiti. There was a dramatic improvement in post-test scores in Haiti (nearly 40%), and this was not seen in Nigeria. Our well-educated cohort with high baseline scores could be a self-selecting group: patients present for clinic visits might have more resources than patients unable to attend clinic visits.^20^ Targeting interventions at newly diagnosed patients may be desirable.^21^

Strikingly, only 51% of patients reported knowing the intent of their treatment. The booklet introduced language (eg, palliative and curative) that is commonplace in oncology, but is usually misunderstood even in monolingual settings and high-income nations.^22,23^ We recommend using phrases that are as explicit as possible, and using local language instead of these terms to reduce confusion.^24,25^

In Nigeria, patients typically present at a later stage.^26,27^ Our sample described cancer predominantly in negative terms, with deadly being the most common word mentioned. Previous qualitative works in Nigeria have highlighted this association of negative emotions and cancer.^28^ Despite the lack of clarity on each patient's treatment intent, curability was also frequently mentioned. A minor, but notable, theme was the financial toxicity of treatment. The cost of care is a driver of cancer outcomes in LMICs.^29,30^

Some limitations of this work include that our sample was more educated than the average Nigerian population, and only included patients with established diagnoses on treatment.^31^ Our post-test was an immediate assessment; so, content retention was not assessed. The post-test was only seven questions and focused primarily on when patients should present to care rather than assessing side effects. Given the data collection methods and multiple parties involved, our data demonstrate highly likely errors and multiple missing data. The English version of the booklet has a Flesch Reading Ease score of 65 (seventh-eighth–grade reading level in the United States).^32^ This reading level is better than many patient facing cancer materials, though is still more difficult to read than is recommended by many organizations.^33,34^ The booklet is picture-based to overcome poor literacy rates. When the booklet is paired with nurses teaching patients, patient understanding appears to be reasonable.^4^ The social desirability response bias could have influenced patient's answers.^35^ Finally, the qualitative analysis is limited by the way responses were recorded. As highlighted above, these are key takeaways for designing future projects.

In conclusion, as access to cancer care expands in LMICs, tools to educate patients are paramount. In this paper, we describe the process of taking an education tool developed in Malawi and Rwanda and adapting it for use in five languages at four cancer centers in Nigeria. We demonstrated that the tool could be delivered by local partners at their own clinics and that the tool was widely recommended to future patients. This sample was well educated and had high pretest scores, limiting our ability to measure the tool's impact on knowledge. We also performed one of the largest qualitative analyses of Nigerian patients with cancer. Given the booklet's acceptability in multiple LMICs, we are looking to further expand access to this resource. The major question moving forward for this booklet, as with any education material, is whether it affects outcomes. Thus, ongoing research to answer this question is warranted.
